# Development and Evaluation of a Molecular Diagnostic Method for Rapid Detection of Histoplasma capsulatum var. farciminosum, the Causative Agent of Epizootic Lymphangitis, in Equine Clinical Samples

**DOI:** 10.1128/JCM.00896-16

**Published:** 2016-11-23

**Authors:** C. E. Scantlebury, G. L. Pinchbeck, P. Loughnane, N. Aklilu, T. Ashine, A. P. Stringer, L. Gordon, M. Marshall, R. M. Christley, A. J. McCarthy

**Affiliations:** a Department of Epidemiology and Population Health, Institute of Infection and Global Health, University of Liverpool, Neston, Wirral, United Kingdom; b Microbiology Research Group, Institute of Integrative Biology, University of Liverpool, Liverpool, United Kingdom; c SPANA Ethiopia, College of Veterinary Medicine and Agriculture, Addis Ababa University, Addis Ababa, Ethiopia; d SPANA UK, London, United Kingdom; e NIHR Health Protection Research Unit in Emerging and Zoonotic Infections, Liverpool, United Kingdom; University of Tennessee

## Abstract

Histoplasma capsulatum var. farciminosum, the causative agent of epizootic lymphangitis (EZL), is endemic in parts of Africa. Diagnosis based on clinical signs and microscopy lacks specificity and is a barrier to further understanding this neglected disease. Here, a nested PCR method targeting the internal transcribed spacer (ITS) region of the rRNA operon was validated for application to equine clinical samples. Twenty-nine horses with signs of EZL from different climatic regions of Ethiopia were clinically examined. Blood samples and aspirates of pus from cutaneous nodules were taken, along with blood from a further 20 horses with no cutaneous EZL lesions. Among the 29 horses with suspected cases of EZL, H. capsulatum var. farciminosum was confirmed by extraction of DNA from pus and blood samples from 25 and 17 horses, respectively. Positive PCR results were also obtained with heat-inactivated pus (24 horses) and blood (23 horses) spotted onto Whatman FTA cards. Two positive results were obtained among blood samples from 20 horses that did not exhibit clinical signs of EZL. These are the first reports of the direct detection of H. capsulatum var. farciminosum in equine blood and at high frequency among horses exhibiting cutaneous lesions. The nested PCR outperformed conventional microscopic diagnosis, as characteristic yeast cells could be observed only in 14 pus samples. The presence of H. capsulatum var. farciminosum DNA was confirmed by sequencing the cloned PCR products, and while alignment of the ITS amplicons showed very little sequence variation, there was preliminary single nucleotide polymorphism-based evidence for the existence of two subgroups of H. capsulatum var. farciminosum. This molecular diagnostic method now permits investigation of the epidemiology of EZL.

## INTRODUCTION

Epizootic lymphangitis (EZL), caused by the dimorphic fungus Histoplasma capsulatum var. farciminosum, is traditionally a disease of equids; the related species H. capsulatum var. capsulatum causes histoplasmosis in humans and is an important opportunistic pathogen worldwide (1). The clinical presentation in horses varies, with four forms being described (2): asymptomatic, ocular, cutaneous, and respiratory. Mixed forms can occur (3). The cutaneous form is characterized by multifocal pyogranulomatous subcutaneous nodules that progress along the lymphatic system, with the coalescence of nodules producing a corded appearance. If lesions are located on the limbs, progression of the disease can result in severe lameness. The respiratory form is classically characterized by pyogranulomatous lesions within the nasal mucosa and lung parenchyma, with potential for multisystemic pathology (2–7).

Although EZL has been eradicated from Europe, it is currently prevalent in Ethiopia, where between 0% and 39% of equids may be infected, with the rate being dependent upon the region (8–10). Ethiopia has Africa's largest equine population with approximately 2 million horses, which have a crucial role in the economy of both urban and rural communities (11–13). In two separate participatory studies in different areas of Ethiopia (14, 15), horse owners consistently volunteered EZL as a high-priority disease. EZL contributes to extensive morbidity and subsequent mortality due to abandonment of chronically infected animals and can have a devastating impact on the incomes of poor families (16, 17).

Within regions where the disease is endemic, access to treatment is a significant challenge. The Society for the Protection of Animals Abroad (SPANA) currently provides free veterinary care within its clinics; however, topical treatment with tincture of iodine and oral dosing with potassium iodides are labor intensive, expensive, and of limited efficacy in moderate to severe cases of EZL (18). It is imperative that animals be diagnosed early in the course of the disease to improve treatment outcomes, conserve resources, and reduce the burden of infection within the population. Currently, due to limited available diagnostic technologies, veterinarians in Ethiopia diagnose the disease on the basis of clinical appearance and microscopic examination for yeast cells within pus. This has the potential for misdiagnosis, as the clinical appearance can mimic that of other diseases (e.g., ulcerative lymphangitis, sporotrichosis, and the cutaneous form of glanders [2, 9, 19, 20]). Culture of H. capsulatum var. farciminosum from clinical lesions would be definitive but is challenging and rarely attempted. Therefore, reliable and robust approaches to diagnosis are required to support clinical decision making and enable epidemiological studies to provide the rationale for the development of disease prevention strategies. EZL has recently been highlighted to be a priority neglected disease of working equids (21).

Classically, members of the genus Histoplasma have been classified into three separate varieties, H. capsulatum var. capsulatum, H. capsulatum var. duboisii, and H. capsulatum var. farciminosum, defined by host species and pathogenesis (1). However, Histoplasma spp. have more recently been grouped into eight clades on the basis of multilocus sequence typing of isolates combined with the geospatial distribution of their sources (22). H. capsulatum var. farciminosum has long been considered an equine-specific pathogen, but the application of molecular biology techniques has identified a broader host and geographic range for H. capsulatum var. farciminosum, with clinical cases being reported in dogs (23), badgers (24), and even humans (25). The phylogeny of Histoplasma spp. has been examined using a range of different gene loci (22, 23, 25–30). As has been frequently reported for fungi, sequence variation in the internal transcribed spacer (ITS) region of the rRNA operon provides a resolving power sufficient to discriminate between closely related species and variants and can contribute to the design of specific PCR-based detection protocols (25, 27, 31, 32). For H. capsulatum var. capsulatum, a nested PCR protocol for the specific identification of the organism in cultures has been designed (31) and was adapted here for use with clinical material. Isolates and/or sequences of both equine and human origin from Africa are underrepresented in studies on the phylogeny of histoplasmas (25, 26, 29, 33–35), which is at odds with their prevalence in these regions (36–39). Phylogenetic analysis of a few historic cultures of specimens from equine clinical cases has been described (25, 27), but no studies have reported the application of PCR to detect H. capsulatum var. farciminosum directly in clinical specimens.

This study had two primary objectives: (i) to establish and validate the use of DNA extraction and PCR amplification protocols to rapidly identify H. capsulatum var. farciminosum directly from equine clinical specimens and stored clinical samples and (ii) to generate ITS region sequences that may provide an insight into strain diversity.

(Summary findings have appeared in published conference abstracts from the British Equine Veterinary Association Annual Conference 2015 [40] and The International Equine Infectious Diseases Conference, Buenos Aires, Argentina, 2016 [41].)

## MATERIALS AND METHODS

The methods for extracting DNA from equine pus were optimized prior to field sampling as follows. DNA extraction was tested on pus samples collected from horses in the UK (incisional site infection and sinusitis samples collected at surgery), and the pus samples were spiked with a 1/10 (vol/vol) cell culture suspension of Saccharomyces cerevisiae as a proxy for H. capsulatum var. farciminosum in order to demonstrate that fungal DNA could be recovered from pus. DNA preparations were obtained using a Qiagen blood and tissue kit according to the manufacturer's instructions, but a starting volume of 50 μl of pus was used and the incubation at 56°C was extended to 2 to 3 h to ensure adequate lysis of the sample. The DNA yield was assessed by use of a NanoDrop spectrophotometer followed by running of the genomic DNA extract on a 2% agarose gel stained with Midori green.

### Case selection and sampling.

Field sampling of clinical cases presented to the SPANA mobile veterinary clinical team was undertaken in Ethiopia between February and April 2014. Cases were selected from each of 7 SPANA clinic sites on the basis of clinical signs suggestive of infection with H. capsulatum var. farciminosum, provided that they presented palpably fluctuant and unruptured nodules. Sampling regions varied by altitude, topography, and climate (see Table S1 in the supplemental material). Verbal informed consent was sought from all participating owners, and the study was approved by the University of Liverpool Research Ethics Committee and the Addis Ababa University College of Veterinary Medicine and Agriculture's ethics board prior to commencement. The horses of all owners attending SPANA clinics received free treatment regardless of their decision to volunteer for the study.

A 10-ml jugular blood sample was taken from each case and placed into EDTA-containing and plain Vacutainer tubes. The area surrounding two unruptured cutaneous nodules was shaved and aseptically prepared, and the contents were aspirated with a 1/2-in. 16-gauge needle. The aspirates were immediately transferred into separate sterile Eppendorf tubes, stored in a cool box prior to transfer to a refrigerator, and processed within 24 h. Horses with respiratory signs were sampled using a nasal swab inserted into the rostral 10 cm of the nasal mucosa. The swabs were placed directly into a universal bottle containing 20 ml sterile saline. Where necessary, horses were sedated to facilitate sampling and allow subsequent treatment.

Blood samples were collected from a further 20 horses from 5 highland regions where few or no cases of EZL had previously been reported (4 horses from each region; see Table S1 in the supplemental material). These horses did not have any cutaneous lesions suggestive of EZL and were randomly selected from a larger cohort of 350 horses being sampled for the presence of respiratory pathogens for comparison with the horses with EZL.

**FIG 1:**
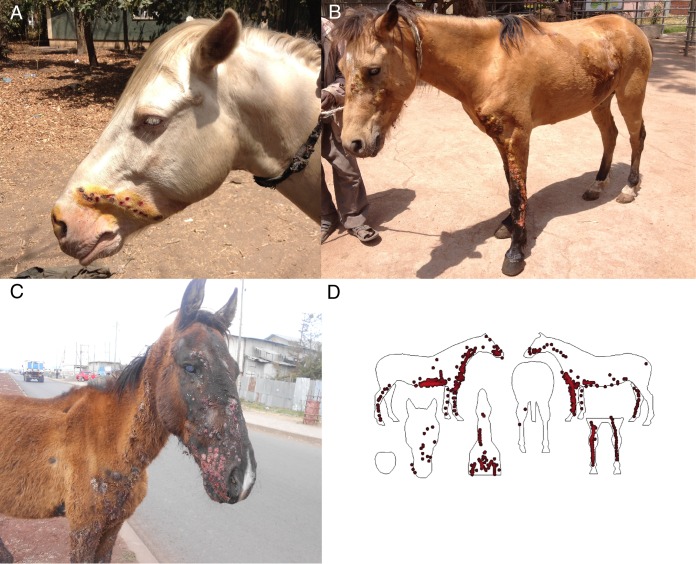
Cutaneous EZL lesions observed in the infected horses. (A) Mild case, in which lesions are evident in only one body area; (B) moderate case, in which lesions are distributed over the left forelimb and other body sites and moderate cording is seen on the forelimb; (C) severe case, in which multiple coalescent nodules appear over all four limbs and extensive lesions appear on the face; (D) spatial distribution of cutaneous lesions of EZL across 29 horses with cutaneous lesions suspected to be EZL plotted onto silhouettes using WebPlotDigitizer software (Ankit Rohatgi; available at http://arohatgi.info/WebPlotDigitizer) and R software (R Foundation for Statistical Computing, Vienna, Austria). The plot demonstrates that cutaneous lesions were more densely distributed around the forelimbs, neck, chest, and girth regions.

Alongside the clinical sampling and treatment, a short questionnaire was delivered to the owners to gather information on the clinical presentation, history of infection, and any previous treatment. The signalment, case presentation, and clinical examination findings (performed by a veterinary surgeon/animal health professional) were recorded, and lesion location was recorded on equine silhouettes (Fig. 1). Suspected EZL cases were categorized as mild, moderate, or severe on the basis of a previously developed grading system (18).

### Sample processing in Ethiopia.

Slide preparations of pus, blood, and nasal swab impression smears were stained with Giemsa using a 1:20 working solution (Giemsa stain preparation; Himedia Labs). The pus samples were examined for the presence of yeast cells suggestive of Histoplasma infection (42) at a ×1,000 magnification under oil immersion. Differential blood cell counts were calculated (43), the hematocrit (packed cell volume) was measured with capillary tubes, and the total protein concentration was measured with a handheld refractometer (44).

Preparations of genomic DNA from pus and blood were made using a Qiagen DNeasy blood and tissue kit (GE Healthcare UK Limited, Buckinghamshire, UK) in the clinical laboratory at SPANA, Debre Zeit, Ethiopia. Blood and 50-μl pus samples were processed according to the manufacturer's protocol. For the lysis step, the pus samples were heated in a water bath at 56°C for 2 to 3 h until the lysate appeared clear. The eluted DNA preparations were dried by evaporation from the Eppendorf tubes at 40°C. Eighty DNA extracts from blood and pus samples were transported to the University of Liverpool under UK (DEFRA)-approved licensing.

### FTA card preparation.

To examine the use of Whatman FTA cards as a convenient method for the capture, transport, and storage of DNA, pus and blood samples from case horses were placed onto classic Whatman FTA indicating cards (GE Healthcare). Aliquots (0.5 ml) of pus diluted with 0.5 ml of sterile saline were inactivated by heat treatment at 95°C for 30 min in a water bath prior to application of 200 μl of sample to the card (45). Two 200-μl blood spots and two heat-inactivated pus samples from each horse were placed onto separate sample zones, and the loaded FTA cards were air dried overnight before being microwaved at full power (800 W) for 30 s, left to stand for 1 min, and microwaved for a further 30 s. A glass beaker containing >200 ml of water was placed alongside the cards in the microwave to dissipate heat. Cards with heat-inactivated samples were individually placed into sealable plastic bags and dispatched to the University of Liverpool, where they were stored at 4°C upon arrival.

### Validation of a nested PCR protocol for detecting H. capsulatum var. farciminosum in DNA preparations.

Preparations of DNA of H. capsulatum var. farciminosum reference strain CBS 539.84 and H. capsulatum var. capsulatum reference strain CBS 137.72 were obtained from the CBS-KNAW Fungal Biodiversity Centre, Netherlands, and used as positive controls. Extracts of Saccharomyces cerevisiae DNA, Escherichia coli DNA, and DNA from pus collected at surgery from a horse in the UK were used as negative controls.

Precipitated DNA samples (Qiagen) were rehydrated with DNA- and RNA-free water and analyzed by use of a NanoDrop spectrophotometer. Aliquots of 50 ng μl^−1^ DNA were prepared and stored at −20°C before use. A nested PCR protocol targeting the ITS region coding for rRNA genes was adapted from that published by Jiang et al. (31). Both sets of primers (P3/2R8 and F5/2R5) spanned the ITS1-5.8S-ITS2 region (31). The optimum annealing temperatures for each primer pair were determined with control H. capsulatum var. farciminosum template DNA. Each reaction mixture contained 50 ng μl^−1^ of template DNA and 10-pmol concentrations of PCR primers P3 and 2R8 added to BioMix red (Bioline Reagents Limited, UK) in a 25-μl reaction volume, as follows: 12 μl BioMix red, 2 μl forward primer and 2 μl reverse primer, 8 μl H_2_O, and 1 μl DNA template. The first-round PCR using P3/2R8 primers (primer P3, 5′-CGGAAGGATCATTACCACGCCG-3′; primer 2R8, 5′-CAGCGGGTATCCCTACCTGATC-3′) was performed with the following thermocycler program: 94°C for 10 min (denaturation) and then 35 cycles of 94°C for 1 min, 49°C for 1 min, and 72°C for 1 min, followed by a final extension cycle of 72°C for 10 min. The product from the first round was expected to be 587 bp, and this was visualized by electrophoresis on a 2% agarose gel stained with Midori green and by comparison to the bands on a 1-kb hyperladder (Bioline). A 1-in-10 (vol/vol) dilution of the product from this first reaction was added to fresh master mix including 10-pmol concentrations of primers F5 and 2R5 (primer F5, 5′-CTACCCGGCCACCCTTGTCTAC-3′ primer 2R5, 5′-CCTACCTGATCCAGTCAACC-3′). The thermocycler program for the second round was the same as that for the first round, except that the annealing temperature was raised to 55°C for 1 min. The expected product was 514 bp and was visualized via electrophoresis at 70 V for 30 min on a 2% (wt/vol) agarose gel stained with Midori green (2 μl per 100 ml agarose). Excess primers and nucleotides were removed from the PCR amplicons using the ExoSAP-IT reagent (USB Products, High Wycombe, UK), and the forward sequence was determined (GATC Biotech AG).

### DNA extraction from pus and blood samples on Whatman FTA cards.

The FTA cards were prepared for analysis using a hole punch (catalog number 9070220; Knippex, Germany). The following protocol produced the most satisfactory DNA yield from both blood and pus samples on Whatman FTA cards and was applied throughout the study. For each pus and blood sample spot, 4 hole punches 5 mm in diameter were produced. Between the collection of each sample, the hole punch was sterilized by immersion in 100% ethanol followed by flaming. The FTA card punch samples were placed into a screw-cap tube containing 0.5 g acid-washed glass beads (diameter, 425 to 600 μm), 0.5 ml H_2_O, and 0.5 ml phenol-chloroform-isoamyl alcohol (25:24:1; pH 8). The solution was mechanically disrupted using a Powerlyser homogenizer at 2,100 rpm for 5 min and then centrifuged at 8,000 × *g* for 3 min. The uppermost aqueous phase was extracted and placed into a sterile tube, and 0.5 ml chloroform was added. The mixture was centrifuged at 8,000 × *g* for 3 min, and the aqueous phase was again collected. DNA was precipitated by adding 1 ml of ethanol and 5 μl of 200 mM sodium acetate and incubating the mixture at −20°C for >2 h. The ethanol precipitate was microcentrifuged at 13,000 × *g* for 10 min, and the supernatant was removed and discarded. The DNA pellet was air dried and then reconstituted in 50 μl sterile DNA- and RNA-free H_2_O. The resulting DNA preparation was analyzed using a NanoDrop spectrophotometer prior to being tested for the presence of H. capsulatum var. farciminosum DNA by nested PCR as described above.

### Cloning PCR products.

A subsample of 9 Histoplasma-positive amplification products from the second round of the nested PCR was cloned in E. coli (46). DNA bands of the expected size from the second round of the nested PCR were excised and extracted using a Bioline Isolate II PCR and gel kit (Bioline). Two microliters of each of the DNA preparations was mixed with 5 μl ligation buffer (60 mM Tris-HCl [pH 7.8], 20 mM MgCl_2_, 20 mM dithiothreitol, 2 mM ATP, 10% polyethylene glycol), 1 μl pGEM-T Easy plasmid vector (Promega Corporation, Madison, WI, USA) containing a gene encoding ampicillin resistance (Promega), and 1 μl a T4 DNA ligase preparation and made up to 10 μl with deionized water. This reaction mixture was then left overnight at 4°C, 4 μl of the ligation reaction mixture was then added to 100-μl aliquots of thawed Escherichia coli TOP10 competent cells, and the ligation reaction mixture and cells were placed on ice for 20 min. The cells were subsequently heat shocked at 42°C for 45 to 50 s and returned to ice for a further 2 min, and 950 μl of LB was then added to each aliquot before incubation at 37°C with shaking for 1.5 h. One hundred microliters of the cell suspension was then pipetted onto LB agar plates containing 100 μg ml^−1^ ampicillin, 100 μl of IPTG (isopropyl-β-d-thiogalactopyranoside; 100 mM), and 20 μl of X-Gal (5-bromo-4-chloro-3-indolyl-β-d-galactopyranoside; 50 mg/ml) for screening for blue and white colonies. White colonies (colonies containing inserts) were then picked, and each was inoculated into 10 ml LB plus 100 μg ml^−1^ ampicillin and incubated for 16 to 24 h at 37°C. Five colonies were randomly selected from each of the 9 samples; the plasmids were then purified from the E. coli cultures (plasmid minikit, isolation of high-copy-number plasmid DNA; Bioline), and the presence of the target DNA in the plasmid was confirmed by amplification with the primer pair F5/2R5, used as described above. Each plasmid DNA preparation was then sequenced (GATC Biotech AG). All sequence data were analyzed using the BLAST search engine (http://blast.ncbi.nlm.nih.gov/Blast.cgi). The cloned sequence data were edited to remove any plasmid DNA, leaving fragments of ca. 514 nucleotides in length encoding the 18S rRNA internal transcribed spacer regions. These fragments were then aligned using ClustalW2 software.

### Screening for Corynebacterium species by multiplex PCR.

As Corynebacterium pseudotuberculosis (which causes ulcerative lymphangitis) infection is one of the differential diagnoses for the cutaneous presentation of EZL (2, 9), all 80 DNA extracts from pus and blood samples were screened by multiplex PCR for the presence of Corynebacterium spp. A freeze-dried culture of Corynebacterium pseudotuberculosis (DSMZ 20689) was obtained from the Leibniz Institute DSMZ (www.dsmz.de/), cultured in brain heart infusion medium with glucose (5 mg ml^−1^), and incubated at 37°C with agitation. DNA was extracted (47), and a multiplex PCR was used to amplify the 16S rRNA gene (816 bp), *rpoB* (encoding the RNA polymerase β subunit; 446 bp), and *pld* (encoding an exotoxin; 203 bp) present in the genome of C. pseudotuberculosis using the six oligonucleotide primers described by Pacheco et al. (48). The amplification products were visualized on a 1% (wt/vol) agarose gel stained with Midori green.

### Statistical analyses.

Descriptive statistics of the clinical examination data were produced, and comparisons between case and control horses were made using SPSS software (version 21; IBM Corporation, USA), with the cutoff for statistical significance being set at a *P* value of <0.05. The results obtained by the different diagnostic methods (nested PCR, microscopy, and pattern recognition of clinical signs) were compared using diagnostic test comparison tables (49). Univariable logistic regression analysis was used to compare clinical signs.

## RESULTS

In total, 29 case horses with suspected EZL and 20 horses, selected for comparison, from highland regions (>2,300 m mean sea level) in Ethiopia where EZL has not previously been reported comprised the study population. This included 28 geldings, 18 stallions, 1 mare, and 2 horses whose status was unrecorded. Table S2 in the supplemental material summarizes the questionnaire and clinical examination findings for all case and control horses. Among the 29 presumptive EZL cases, 11 (38%) were classified as having early EZL, 12 (41%) were classified as having moderate EZL, and 6 (21%) were classified as being severely affected cases (Fig. 1).

Three of the case horses had signs of lower respiratory disease: 1 was categorized as having mild EZL with lesions on the lateral neck and generally increased respiratory noise detected on thoracic auscultation, 1 was categorized as having moderate EZL and observed to be coughing with a bilateral mucopurulent nasal discharge and retropharyngeal lymph node swelling, and 1 was categorized as having severe EZL with a bilateral mucopurulent nasal discharge and ulceration apparent on the nasal mucosa. Nasal swab specimens were collected from the last 2 horses and tested positive for H. capsulatum var. farciminosum by the nested PCR diagnostic method reported here. A further seven case horses presented with a range of mild upper respiratory signs.

Five of the case horses showed various degrees of lameness, and nine had ocular abnormalities present. No typical ocular signs of EZL lesions were seen among these cases, as has been indicated previously (2, 3). EZL case horses tended to demonstrate eosinopenia and monocytosis compared to the controls (*P* < 0.05) (see Table S2 in the supplemental material), which is consistent with a chronic pyogranulomatous infection.

### Diagnostic tests.

**FIG 2:**
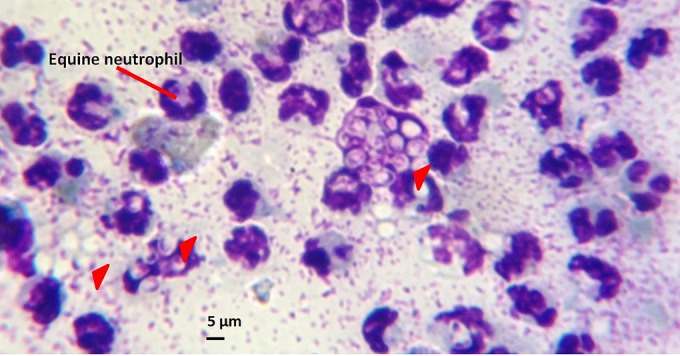
Light micrograph of a pus impression smear from an EZL case horse stained with Giemsa and examined for the presence of Histoplasma yeast cells. The impression smear of pus aspirated from an unruptured subcutaneous nodule was viewed at ×1,000 magnification. Arrowheads, clusters of ovoid to lemon-shaped yeast cells (diameter, 4 to 5 μm) with a characteristic refractive cell wall. For comparison, an equine neutrophil is approximately 12 to 15 μm in diameter.

Impression smears were prepared from a total of 27 pus samples and 28 blood samples from the 29 case horses (in 2 horses with cutaneous lesions, it was not possible to aspirate nodules, and for 1 horse, only a pus sample was taken). Yeast cells were apparent in 14 (52%) pus smear preparations, as determined by light microscopy (Fig. 2). No yeast cells were visible on any of the blood smear preparations.

A total of 80 Qiagen DNA extractions were made from blood, pus, and nasal swab specimens from 29 case horses (duplicate extractions were prepared for the majority of horses). The overall DNA yield varied between samples, but the median was 68 ng μl^−1^ and the *A*_260_/*A*_280_ ratio was 1.8. The DNA yields from the FTA card samples processed by the phenol-chloroform extraction method had a median yield of 57 ng μl^−1^ and an *A*_260_/*A*_280_ ratio of 1.2.

Samples were scored positive if the nested PCR protocol produced 514-bp amplicons, as visualized by agar gel electrophoresis. In all cases, nested PCR was found to be necessary, and amplification products could not be reliably detected after the initial round of amplification with the P3/2R8 primer pair. Similarly, primary amplification with the F5/2R5 primer pair did not yield products, nor did two rounds of amplification with these primers. Control DNA from H. capsulatum var. capsulatum and H. capsulatum var. farciminosum cultures could be amplified with the individual primer pairs when they were used directly, but this was not nearly as reliable as the nested PCR protocol. The detection limits of the nested PCR were tested using serial dilutions of the H. capsulatum var. capsulatum control DNA template. At a 1/100 dilution, it was still possible to detect 0.5 ng H. capsulatum var. capsulatum DNA using this nested PCR.

**FIG 3:**
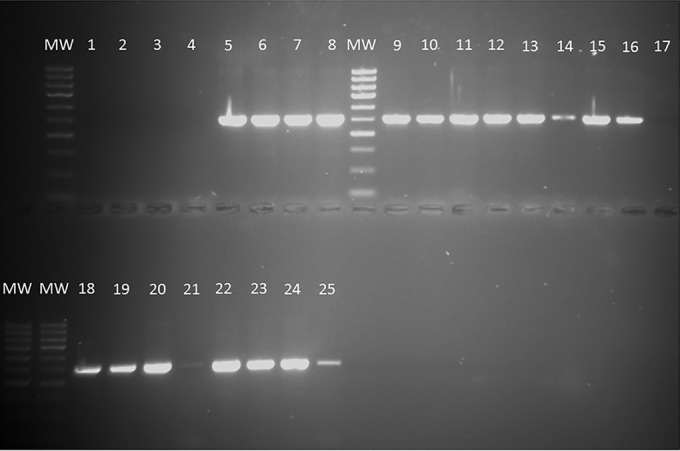
Gel electrophoresis of nested PCR amplification products obtained from DNA extracted from horse pus and blood samples. DNA preparations were amplified with primers P3/2R8 (first round) and then diluted 1 in 10 and subjected to a second round of PCR amplification with PCR primers F5/2R5 to generate ITS gene products (514 bp) indicative of the presence of Histoplasma DNA. All amplification products were subsequently sequenced to confirm >97% identity and the closest match to Histoplasma capsulatus ITS region DNA. Lanes 1 to 4, negative controls (DNA extracts from S. cerevisiae, E. coli, pus from a horse in the UK, and DNA- and RNA-free water, respectively); lane 5, H. capsulatum var. capsulatum control DNA; lane 6, H. capsulatum var. farciminosum control DNA; lanes 7 to 25, PCR amplicons of Qiagen DNA extracts of blood and pus from horses with suspected EZL (lanes 7 to 23, DNA extracts from pus, lanes 24 and 25, DNA extracts from blood); lanes MW, molecular weight markers).

In total, 25 of the 27 EZL case horses tested positive on the basis of analysis of Qiagen DNA extracts from pus, and 17 of the 27 (63%) EZL case horses tested positive on the basis of analysis of Qiagen DNA extracts from blood samples; example results are presented in Fig. 3. Of the FTA card samples, 24 of the 27 pus samples and 23 of the 28 blood samples were positive for H. capsulatum var. farciminosum, as determined by the nested PCR protocol. All these results compare very favorably with the low levels of detection of yeast cells (52%) in pus samples taken from these horses with EZL. Determination of the forward sequence of all nested PCR amplification products confirmed their identity as histoplasmas (≥97% similarity by BLAST analysis; data not shown). Of the FTA card blood samples from the 20 control horses (horses originating from outside areas where EZL is endemic and showing no signs of disease), two samples had positive results by nested PCR (see Table S3 in the supplemental material). Sequencing also confirmed that these amplicons were from Histoplasma DNA. While the PCR results obtained from the Qiagen extracts were reproducible for each of the 10 horses for which tests were repeated, the PCR results were not reproducible for all of the pus and blood spots presented on FTA cards (see Table S3); 4 of the 27 pus samples and 9 of the 28 blood samples gave positive results that were not completely reproducible. Therefore, for the purposes of data analysis, we identified a horse to be positive for histoplasmosis if at least one of the pus samples or at least one of the blood samples was positive for H. capsulatum var. farciminosum on nested PCR.

In addition to simple sequencing of potentially mixed PCR amplification products to confirm the identity of amplicons as Histoplasma amplicons, we cloned 15 of these to produce a total of 43 clone sequences of the amplified ITS region for more detailed phylogenetic analysis. Bona fide sequence data were obtained for 38 full-length 514-bp amplicons, and these were aligned (see Fig. S1 in the supplemental material).

**FIG 4:**
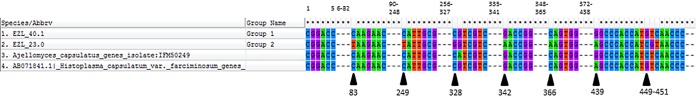
Sequence alignment of 514-bp cloned fragments of the Histoplasma ITS region. The diagram illustrates the nine consistent SNPs (indicated by arrowheads), identified here, that divide the H. capsulatum var. farciminosum ITS clones (38 clones) into two subgroups. Subgroup 1 comprised 23 of the clones, and subgroup 2 comprised the remaining 15. The numbers at the top are the nucleotide positions in the ITS region. The two reference sequences of H. capsulatum var. capsulatum (Ajellomyces capsulatus) and H. capsulatum var. farciminosum were downloaded from GenBank.

Across the entire 514-bp fragment of the ITS region sequenced here, there were nine consistent single nucleotide polymorphisms (SNPs; representing a 1.8% substitution rate) that divided the 38 clone sequences into two coherent subgroups. These were TC at position 83, TC at position 249, GC at position 328, AG at position 342, AC at position 366, AG at position 439, and 3 sequential nucleotide substitutions (CGT in place of GTC) at positions 449 to 451. The alignment of these two subgroups and the comparable sequences of H. capsulatum var. capsulatum and H. capsulatum var. farciminosum are presented in Fig. 4. Representatives of each subgroup occasionally originated from a sample from a single horse, and analysis of the contextual metadata did not reveal any patterns in the origin of the samples or the stage of disease.

Microscopy generally had a lower sensitivity and specificity than PCR for both Qiagen and FTA card extracts (Table 1). In all cases where yeast cells were visible on microscopy, the PCR test was positive with pus samples, except in one instance among the FTA card preparations. Yeast cells were never observed microscopically in blood samples; however, H. capsulatum var. farciminosum was detected by nested PCR in many of these samples (16 positive samples among Qiagen extracts and 21 positive samples among FTA card preparations; Table 2; see also Table S3 in the supplemental material). Among this case series, if clinical signs were apparent, there was a >80% probability that the diagnosis would be confirmed by PCR of blood samples (Table 3). None of the 80 Qiagen DNA extracts from blood and pus samples tested positive for Corynebacterium spp. with the multiplex PCR diagnostic test (48) applied here, validated by the inclusion of appropriate positive and negative controls.

**TABLE 1:** Comparison of sensitivity of detection of H. capsulatum var. farciminosum by microscopy and nested PCR with DNA extracts from Qiagen and FTA card preparations of pus and blood from horses suspected of clinical EZL^*a*^

Methods compared	Sensitivity	Specificity	PPV	NPV
Microscopy and PCR with Qiagen pus extracts	0.56	1	1	0.15
Microscopy and PCR with Qiagen blood extracts	0.44	0.22	0.5	0.18
Microscopy and PCR of pus on FTA card prepn	0.5	0.5	0.92	0.08
Microscopy and PCR of blood on FTA card prepn	0.52	0.4	0.79	0.17

a Sensitivity describes the proportion of microscopy-positive samples in which H. capsulatum var. farciminosum was correctly detected compared with the PCR results. Specificity describes the proportion of microscopy-negative samples in which H. capsulatum var. farciminosum was correctly detected compared with the PCR results. PPV, positive predictive value of microscopy compared to the results of PCR, or the probability of a positive result on microscopy given that the test is positive by PCR. NPV, negative predictive value of microscopy compared to the results of PCR, or the probability of a negative result on microscopy given that the PCR result is negative.

**TABLE 2:** Comparison of microscopic visualization of yeast cells in pus with nested PCR detection using DNA extracts from Qiagen and FTA card preparations of pus and blood from horses suspected of clinical EZL^*a*^

Yeast cells in pus on microscopy	No. of samples with the indicated PCR result^*b*^ with Qiagen extracts from:				No. of samples with the indicated PCR result with an FTA card prepn of:			
	Pus		Blood		Pus		Blood spot	
	Positive	Negative	Positive	Negative	Positive	Negative	Positive	Negative
Present	14	0	7	7	12	1	11	3
Absent	11	2	9	2	12	1	10	2
Total	25	2	16	9	24	2	21	5

a The nested PCR test result is considered the gold standard here because a known H. capsulatum var. farciminosum control was used in the test. No blood was taken from 1 horse with clinical signs suspicious of EZL; one horse did not have a Qiagen DNA extract made from a blood sample.

b PCR test results were based on at least one positive PCR test result per horse.

**TABLE 3:** Comparison of diagnosis based on the presence or absence of clinical signs of EZL with the results of nested PCR with blood spots on FTA card preparations from 48 horses^*a*^

Presumptive EZL on the basis of clinical signs	No. of samples with the following PCR result with blood on FTA card prepn^*b*^:	
	Positive	Negative
Yes	23	5
No	2	18
Total	25	23

a The 48 horses included 28 with clinical signs of EZL and 20 with no clinical signs. The nested PCR test result is considered the gold standard here because a known H. capsulatum var. farciminosum control was used in the test. When the results of diagnosis based on clinical signs were compared with the results of PCR, the sensitivity was 0.92, the specificity was 0.78, the positive predictive value was 0.82, and the negative predictive value was 0.9, where sensitivity, specificity, positive predictive value, and negative predictive value are defined in footnote *a* of Table 1.

b PCR test results are based on at least one positive PCR test result per horse.

## DISCUSSION

The nested PCR primer sets developed by Jiang et al. (31) for Histoplasma capsulatum var. capsulatum cultures were used here to develop a diagnostic test for H. capsulatum var. farciminosum infection in clinical samples from horses. Two-stage nested amplification was found to be necessary, and this may have been due to the biological diversity within the clinical samples, which contained a variety of equine DNA, including DNA from immune cells, degradation factors, and potentially low numbers of yeast cells. This is comparable to other scenarios where nested PCRs are required to detect fungal targets in complex clinical samples (50). The diagnostic reliability of the nested PCR protocol was much superior to that of conventional microscopy or the sole reliance on clinical signs. In all cases, sequencing of the 514-bp amplicons demonstrated the presence of Histoplasma DNA, and this was further confirmed by sequencing of a large sample of clones. PCR methods targeting the ITS region have previously been used to identify H. capsulatum var. capsulatum (27, 29) and have the additional advantage of enabling strain sequence variation to be explored (31). In that respect, we also identified a collection of single nucleotide polymorphisms (SNPs) that suggest a delineation of at least two subgroups of H. capsulatum var. farciminosum circulating in the working horse population in Ethiopia. At this early stage, there is no evidence that the occurrence and distribution of these two subgroups correlate with clinical signs and the severity of disease, geographical location, sample source, or any other identifiable parameter. In fact, both sequence variants were recovered from the same animal on a few occasions. Alignment of H. capsulatum var. farciminosum and H. capsulatum var. capsulatum ITS region sequences showed a high degree of conservation, in agreement with the minimal diversity that was reported among Histoplasma spp. in the ISHAM ITS barcoding project (32), so it may be more appropriate to target alternative genetic loci in histoplasmas in any search for markers of variation that may have epidemiological utility. Previous molecular taxonomic studies have identified a close link between H. capsulatum var. capsulatum and H. capsulatum var. farciminosum (25, 26), and therefore, characterization of strains from current regions of endemicity is appropriate. There are many unanswered questions; e.g., can H. capsulatum var. farciminosum be zoonotic, is there any strain variation that impacts virulence and clinical presentation, is there host specificity, what are the modes of transmission, are vectors involved, and what is the epidemiology of EZL? The biological relationship and functional difference between H. capsulatum var. capsulatum, a relatively well studied and principally human pathogen, and H. capsulatum var. farciminosum, primarily a specific pathogen of equids, are unknown. Representative strains of Histoplasma spp. (including H. capsulatum var. capsulatum and H. capsulatum var. farciminosum) from regions of endemicity on the African continent are especially lacking, and this is a public health blind spot in view of the high burden of infection (36–39).

Another important aspect of this study is the demonstration that the nested PCR assay can be used to detect H. capsulatum var. farciminosum in inactivated pus and blood samples dried onto Whatman FTA cards. These cards have been used extensively to archive a range of human, animal, plant, bacterial, fungal, and viral pathogens and are a convenient and cost-effective method to store DNA and RNA for downstream molecular applications without the need for specialist equipment (45, 51, 52). Here, the advantages of Whatman FTA cards for the collection of samples in the field, for the inactivation of pathogens, and for the transport and storage of samples of equine pus and blood for application of molecular diagnostic methods are obvious. They are very suitable for use in countries with limited resources, but ideally, we would hope to simplify the DNA extraction protocol especially by finding an alternative to phenol-chloroform treatment, which was used because it was found to be very effective compared to a number of other methods tested. The stability of DNA in equine pus and blood samples stored on FTA cards also needs to be established; previous studies have identified issues concerning storage and sample longevity (53).

This is the first report of the PCR-based detection of H. capsulatum var. farciminosum directly in equine blood. PCR detection of Histoplasma spp. directly from human blood samples has been reported previously (54–56), and a fungemic stage is recognized in human infection (57, 58). Our data suggest the identification of H. capsulatum var. farciminosum fungemia in horses, although further work is required to determine the timing of the development of fungemia and investigate the potential for the early detection of H. capsulatum var. farciminosum directly from blood samples. Furthermore, our identification of H. capsulatum var. farciminosum DNA in two blood samples from horses with no clinical signs of EZL could be due to subclinical infection and/or asymptomatic carriage, both aspects of EZL about which there is currently no information.

Ideally, EZL should not be diagnosed in horses in Ethiopia and other countries where EZL has been reported solely on the basis of clinical signs. There are a number of other pathogens causing infections which have similar clinical presentations. Although we were able to exclude ulcerative lymphangitis by demonstrating that all 29 horses were PCR positive for H. capsulatum var. farciminosum and simultaneously negative for Corynebacterium spp., we did not address sporotrichosis (59) and the cutaneous (farcy) form of glanders (20), both of which can easily be confused with EZL. Seroconversion in response to glanders exposure has recently been reported among donkeys within similar regions of Ethiopia (60), which further emphasizes the importance of establishing the causative agent of disease in individual horses that present with ulcerative and pyogranulomatous lesions.

Histoplasmosis is a problem not only among horses but also among human populations, where H. capsulatum var. capsulatum has been recognized as a severely underdiagnosed chronic disease (36, 37, 61–63). Among a large case series in people, it was reported that a spectrum of presenting clinical signs could be associated with histoplasmosis, and this had implications for delays in clinical recognition, requests for suitable diagnostic tests and the initiation of trial treatment, and ultimately, case outcome (64). Likewise, lesion location (Fig. 1) and the clinical presentation of EZL varied widely among the equine cases presented here, and H. capsulatum var. farciminosum was also identified in DNA extracts from nasal swab samples from two of the most severe cases with respiratory signs. The well-characterized human variant, H. capsulatum var. capsulatum, is predominantly a respiratory pathogen (36), whereas the role of H. capsulatum var. farciminosum as a respiratory pathogen in horses is largely unknown (2, 4, 7, 65).

A recent workshop on infectious diseases of working equids called for “increased research to address technical data gaps, advocacy to secure funding, and improved surveillance at national and international level to allow further understanding of pathogenesis, diagnosis, treatment and prevention of disease such as epizootic lymphangitis” (21). Robust, reliable, and rapid diagnostic tools are essential both for decision making by veterinarians and to enable large-scale studies that will allow further understanding of the epidemiology, ecology, and transmission of this neglected disease. Ideally, rapid diagnostic techniques could be beneficially utilized within regions of endemicity, and therefore, future work should focus upon examining the practicalities and potential for transfer of technology to regional laboratories, academics, and clinicians within Ethiopia and other regions. Resources to perform PCR are currently available in only some research laboratories within Ethiopia, where diagnostics could be further developed. A WHO report investigating research facilities in national health research systems in sub-Saharan Africa stated that “major constraints are dominated by financial input for equipment purchase, maintenance and laboratory supplies” (66). These factors need to be addressed in order to support and act upon future research developments within regions where the disease is endemic.

## Supplementary Material

Supplemental material
