# Supplementary material for: Development and Evaluation of a Molecular Diagnostic Method for Rapid Detection of Histoplasma capsulatum var. farciminosum, the Causative Agent of Epizootic Lymphangitis, in Equine Clinical Samples
Source: J Clin Microbiol. 2016 Nov 23;54(12):2990–9. doi: 10.1128/JCM.00896-16 (PMC5121390; doi:10.1128/JCM.00896-16)
Supplement: Supplemental material [file supp_54_12_2990__index.html]

Supplemental material 

# Development and Evaluation of a Molecular Diagnostic Method for Rapid Detection of Histoplasma capsulatum var. farciminosum, the Causative Agent of Epizootic Lymphangitis, in Equine Clinical Samples

## Supplemental material

**Files in this Data Supplement:**

- Supplemental file 1 -

  Fig. S1 (Alignment of all 38 cloned fragments of the amplified ITS region showing the 9 consistent SNPs along the 514-bp region)

  PDF, 295K
- Supplemental file 2 -

  Tables S1 (Origins of horses and estimated regional prevalence of EZL in Ethiopia), S2 (Summary of questionnaire responses, clinical observations, and haematology findings of case and control horses), and S3 (Complete data set showing each horse, town of origin, EZL severity category, and PCR-based detection results obtained from repeat testing on Qiagen and FTA card samples of blood and pus) and Fig. S1 legend

  PDF, 434K
