# Supplementary material for: Development and Evaluation of a Molecular Diagnostic Method for Rapid Detection of Histoplasma capsulatum var. farciminosum, the Causative Agent of Epizootic Lymphangitis, in Equine Clinical Samples
Source: J Clin Microbiol. 2016 Nov 23;54(12):2990–9. doi: 10.1128/JCM.00896-16 (PMC5121390; doi:10.1128/JCM.00896-16)
Supplement: Supplemental material [file JCM.00896-16_zjm999095239so1.pdf]

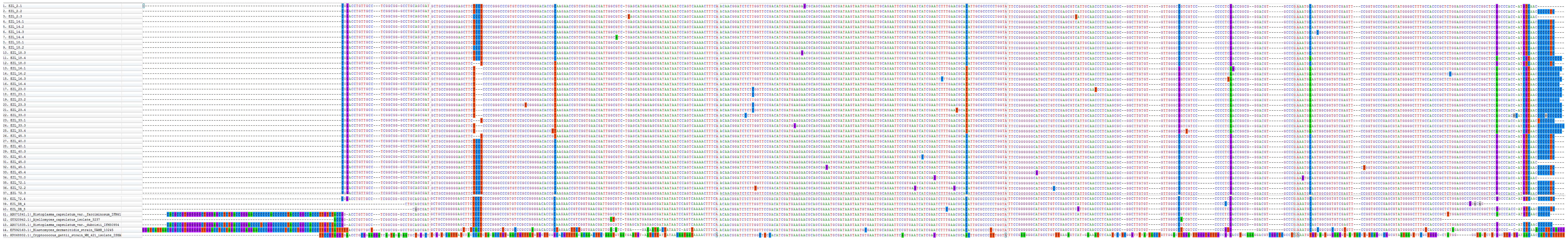
1. EZL_2.1
2. EZL_2.2
3. EZL_2.3
4. EZL_14.1
5. EZL_14.2
6. EZL_14.3
7. EZL_14.4
8. EZL_18.1
9. EZL_18.2
10. EZL_18.3
11. EZL_18.4
12. EZL_18.0
13. EZL_16.1
14. EZL_16.2
15. EZL_16.3
16. EZL_16.4
17. EZL_23.0
18. EZL_23.1
19. EZL_23.2
20. EZL_23.3
21. EZL_23.4
22. EZL_33.0
23. EZL_33.1
24. EZL_33.3
25. EZL_33.4
26. EZL_45.3
27. EZL_40.0
28. EZL_40.1
29. EZL_40.3
30. EZL_40.4
31. EZL_45.0
32. EZL_45.1
33. EZL_45.4
34. EZL_70.0
35. EZL_72.1
36. EZL_72.2
37. EZL_72.3
38. EZL_72.4
39. EZL_DB_4
40. EZL_DB_8
41. AB071841.1|_Histoplasma_capsulatum_var._farciminosum_IFM41
42. GU320942.1|_Ajellomyces_capsulatus_isolate_3237
43. AB071835.1|_Histoplasma_capsulatum_var._duboisii_IFM50954
44. EF592163.1|_Blastomyces_dermatitidis_strain_UAMH_10245
45. KP068802.1|_Cryptococcus_gattii_strain_WM_421_isolate_ISHA
