# Supplementary material for: Development and Evaluation of a Molecular Diagnostic Method for Rapid Detection of Histoplasma capsulatum var. farciminosum, the Causative Agent of Epizootic Lymphangitis, in Equine Clinical Samples
Source: J Clin Microbiol. 2016 Nov 23;54(12):2990–9. doi: 10.1128/JCM.00896-16 (PMC5121390; doi:10.1128/JCM.00896-16)
Supplement: Supplemental material [file JCM.00896-16_zjm999095239so2.pdf]

Supplementary material for

Development and evaluation of a molecular diagnostic method to rapidly detect *Histoplasma capsulatum var. farciminosum*, the causative agent of Epizootic Lymphangitis, in equine clinical samples.

Scantlebury, C.E.[a], Pinchbeck, G.L.[a], Loughnane, P.[b], Aklilu, N.[c], Ashine, T.[c], Stringer, A.P.[d], Gordon, L.[b], Marshall, M.[b], Christley, R.M.[a], McCarthy, A.J[b].

Table S1: Origins of horses (cases and controls) and estimated regional prevalence of EZL in Ethiopia.

Town	Altitude (metres above sea level)	Location N/E	Case or control	Number of horses sampled	Previously reported prevalence of EZL in region.
Akaki	2036	8.87/38.79	Case	7	Period prevalence 4.4%[1]
Debre Zeit	1803	8.59/39.13	Case	8	Period prevalence 10.1%[1] 30%[2]
Hawassa	1702	7.06/38.48	Case	1	12.3%[2]
Legotaffa	N/A	N/A	Case	1	Not reported
Modjo	1785	8.60/39.13	Case	1	39%[2]
Nazreth	1712	8.52/39.27	Case	9	21%[2]
Shashamene	1920	7.21/38.59	Case	2	18%[2]
Wenchi	3115	8.79/37.88	Control	4	Not reported
Debre Brehan	2834	9.68/39.54	Control	4	0%[2]
Salali	2643	9.62/38.94	Control	4	Not reported
Asasa	2360	7.11/39.20	Control	4	7.5%[2]
Chefe	2424	8.97/39.12	Control	4	Not reported

Table footnote: Based on clinical signs and confirmed with either culture or microscopic examination of pus smear. (References [1] Endebu, B. & Roger, F. (2003) International Journal of Applied Research in Veterinary Medicine **1** (3) / [2] Ameni, G. 2006 The Veterinary Journal **172** (1): 160-165). N/A, not available.

Table S2: Summary of questionnaire responses, clinical observations and haematology findings of case and control horses

	Normal Reference Ranges[1]	CASE horses n = 29	CONTROL n = 20 horses	Univariable analysis comparing cases and controls (log-likelihood test)
Age based on dental estimation		Median 10 years (IQ range 5.3; min 5, max 15 years)	Mean 12 years (IQ range 5.3; min 3.5, max 15 years)	p=0.1
Duration of ownership		Median 9 months (IQR 7 months; min 1, max 36 months)	Mean 27 months (IQR 49; min 5, max 73 months)	p=0.005*
Duration of current infection		Median 2 months (IQR 1.4; min 0.2, max 5 months)	N/A	
Number of horses previously treated		8	N/A	
Number of horses still working		21	20	
Number of horses sharing equipment		15	N/A	
Body temperature (^{o}C)	37.2-38.3	Mean 37.9 (IQR 1.1; min 36.7, max 39.0)	Mean 36.5 (IQR 1.1; min 35.3, max 37.9)	p=0.01*
Pulse (bpm)	28-44	Median 48 (IQR 12; min 32, max 64)	Median 48 (IQR 12; min 40, max 60)	p=0.4
Respiration (rpm)	10-24	Mean 28 (IQR 12; min 16, max 56)	Median 38 (IQR 16; min 20, max 60)	p=0.008*
Packed Cell Volume (PCV %)	32-48	Mean 31 (IQR 7.5; min 24, max 44)	Mean 34 (IQR 4; min 26, max 45)	p = 0.3
Total protein in blood (gdL^{-1})	6.0-8.5	Median 7.9 (IQR 1.1; min 6.4, max 10.0)	Median 8.0 (IQR 1.1; min 7.2, max 9.2)	p = 0.7
Neutrophils % (including bands and segmented)	30-75	Median 48 (IQR 26; min 22, max 73)	Median 48 (IQR 18; min 36, max 80)	p = 0.9
Eosinophils %	1-8	Median 3 (IQR 2; min 0, max 9)	Median 4 (IQR 6; min 0, max 16)	p = 0.04*
Basophils %	0-3	Median 0 (IQR 0; min 0, max 2)	Median 0 (IQR 0; min 0, max 1)	p = 0.6
Lymphocytes %	25-60	Median 35 (IQR 21; min 18, Max 63)	Median 38 (IQR 12; min 14, max 62)	p = 0.6
Monocytes %	1-8	Median 8 (IQR 4; min 2, max 18)	Median 2 (IQR 2.5; min 0, max 4)	p = 0.005*

No signs of infection were reported for other horses in contact with study cases. Horses that had previous treatment had mostly been treated with tincture of iodine / oral potassium iodide; some horses had received home based treatment that included application of engine oil / battery acid.

*Statistical significance set at $p<0.05$

[1]Reference ranges from Cowell and Tyler 1992, Cytology and Hematology of the Horse, American veterinary Publications Inc. pp. 191-207.

N/A, not applicable.

Table S3 – Complete dataset showing each horse, town of origin, EZL severity category, and PCR based detection results obtained from repeat testing on Qiagen and FTA card samples of blood and pus. A horse was identified as positive if at least one PCR test was positive (1) ; 0 indicates negative. The number of times the PCR test was repeated on the same horse sample is indicated by the number of + and/or – signs.

Horse number	Date sample collected	Town of Origin	EZL Disease category	Locomotor	Respiratory	Ocular	Yeast visible on pus smear	PCR+ Qiagen pus	Repeatability of PCR Qiagen Pus	PCR+ FTA pus	Repeatability of PCR FTA pus	PCR+ Qiagen Blood	Repeatability of PCR Qiagen Blood	PCR+ FTA blood	Repeatability of PCR FTA blood
1	14.02.14	Akaki	Moderate	NAD	NAD	Bilateral mucoid discharge	0	1	+++	1	+++++++	1	+	1	+ -
2	14.02.14	Akaki	Mild	NAD	Watery nasal discharge	NAD	NS	NS	NS	NS	NS	1	+	1	- +
3	14.02.14	Akaki	mild	NAD	NAD	NAD	0	1	+	1	++++	1	+	1	++
4	14.02.14	Akaki	Mild	NAD	NAD	Ocular discharge	NS	NS	NS	NS	NS	0	-	1	+
5	14.02.14	Akaki	Moderate	NAD	NAD	NAD	0	1	+	1	-+++	1	+	1	--
6	17.02.14	Debre Zeit	Moderate	NAD	NAD	NAD	1	1	++	1	++++	0	-	1	++
7	17.02.14	Debre Zeit	Moderate	NAD	NAD	NAD	1	1	+	1	-+-	1	+	1	-+
8	18.02.14	Nazret	Mild	NAD	NAD	Bilateral mucoid ocular discharge	0	1	+	1	+--	0	-	1	++
9	18.02.14	Nazret	Mild	NAD	NAD	NAD	1	1	+	1	+++	0	-	1	-+
10	19.02.14	Modjo	Moderate	NAD	NAD	NAD	1	1	+	1	+	0	-	1	+-
11	20.02.14	Debre Zeit	Mild	NAD	NAD	NAD	0	1	++	1	+	1	+	0	---
12	20.02.14	Debre Zeit	Euthanasia Moderate	NAD	Bilateral mucopurulent nasal discharge no lung sounds	Left eye corneal opacity	1	1	+++++++	1	+	1	+	1	-+
13	21.02.14	Akaki	Mild	Lameness	Increased respiratory noise (no wheezing / moist sounds)	NAD	1	1	+	ND	ND	1	+	1	++
14	21.02.14	Akaki	Moderate	NAD	Bilateral serous nasal discharge	NAD	1	1	++	1	+	0	-	1	++
15	21.02.14	Debre Zeit	Euthanasia Severe	Lameness	NAD	NAD	1	1	+	1	+	1	+	1	-
16	21.02.14	Legotaffa	Euthanasia Severe	NAD	NAD	Bilateral corneal opacity	0	1	+++	1	++	0	-	1	+
17	24.02.14	Debre Zeit	Mild	NAD	NAD	NAD	1	1	+	0	-	0	-	0	-

Horse number	Date sample collected	Town of Origin	EZL Disease category	Locomotor	Respiratory	Ocular	Yeast visible on pus smear	PCR+ Qiagen pus	Repeatability of PCR Qiagen Pus	PCR+ FTA pus	Repeatability of PCR FTA pus	PCR+ Qiagen Blood	Repeatability of PCR Qiagen Blood	PCR+ FTA blood	Repeatability of PCR FTA blood
18	24.02.14	Debre Zeit	Moderate	NAD	Coughing, bilateral mucopurulent discharge, retropharangeal lymph node swelling	NAD	0	1	+++	1	+++	1	++	0	-
19	24.02.14	Debre Zeit	Severe	NAD	NAD	NAD	1	1	+++++	1	+++++	1	+	1	+
20	25.02.14	Nazret	Mild	NAD	NAD	NAD	0	0	-	1	+	NS	NS	NS	NS
21	25.02.14	Nazret	Respiratory Severe	NAD	Respiratory form of EZL bilateral mucopurulent discharge, ulcers on nasal mucosa, swollen sub mandibular lymph nodes	Blind in left eye, phthitic eye, right eye NAD	0	1	++	0	--	ND	ND	1	+
22	25.02.14	Nazret	Severe	NAD	Watery nasal discharge	NAD	1	1	+++++	1	++++-	1	+	0	-
23	25.02.14	Nazret	Moderate	NAD	NAD	Serous ocular discharge from right eye and swollen conjuntivae	0	1	+	1	+	1	+	1	+
24	13.03.14	Shashamene	Mild	NAD	NAD	Watery ocular discharge from right eye	1	1	+	1	++	0	-	1	+-
25	01.04.14	Nazret	Moderate	NAD	NAD	Lacrimation of right eye only	0	1	+	1	++	1	+	1	+-
26	01.04.14	Nazret	Mild	NAD	Watery bilateral nasal discharge	NAD	0	0	-	1	++	1	+	1	++
27	01.04.14	Nazret	Moderate	Lameness right hind limb	Unilateral nasal discharge	NAD	1	1	+	1	++	0	-	0	--
28	11.03.14	Hawassa	Severe	One forelimb and one hind limb lameness	NAD	NAD	0	1	+	1	++	1	+	1	++

Horse number	Date sample collected	Town of Origin	EZL Disease category	Locomotor	Respiratory	Ocular	Yeast visible on pus smear	PCR+ Qiagen pus	Repeatability of PCR Qiagen Pus	PCR+ FTA pus	Repeatability of PCR FTA pus	PCR+ Qiagen Blood	Repeatability of PCR Qiagen Blood	PCR+ FTA blood	Repeatability of PCR FTA blood
29	13.03.14	Shashamene	moderate	Lame forelimb	Watery nasal discharge	NAD	1	1	+	1	++	1	+	1	+-
C1	17.09.13	Salali 4	none	*	NAD	*	ND	*	*	*	*	*	*	0	--
C2	17.09.13	Salali 8	none	*	NAD	*	ND	*	*	*	*	*	*	0	--
C3	17.09.13	Salali 12	none	*	NAD	*	ND	*	*	*	*	*	*	0	--
C4	17.09.13	Salali 16	none	*	NAD	*	ND	*	*	*	*	*	*	0	--
C5	21.09.13	Chefe 4	none	*	NAD	*	ND	*	*	*	*	*	*	0	--
C6	21.09.13	Chefe 8	none	*	NAD	*	ND	*	*	*	*	*	*	0	--
C7	21.09.13	Chefe 12	none	*	NAD	*	ND	*	*	*	*	*	*	0	--
C8	21.09.13	Chefe 16	none	*	NAD	*	ND	*	*	*	*	*	*	0	--
C9	26.10.13	Wenchi 4	none	*	NAD	*	ND	*	*	*	*	*	*	0	--
C10	26.10.13	Wenchi 8	none	*	NAD	*	ND	*	*	*	*	*	*	0	--
C11	26.10.13	Wenchi 12	none	*	NAD	*	ND	*	*	*	*	*	*	0	--
C12	26.10.13	Wenchi 16	none	*	NAD	*	ND	*	*	*	*	*	*	0	--
C13	02.10.13	**Debre Brehan 4**	none	*	NAD	*	ND	*	*	*	*	*	*	**1**	**++**
C14	02.10.13	**Debre Brehan 8**	none	*	NAD	*	ND	*	*	*	*	*	*	**1**	**++**
C15	02.10.13	Debre Brehan 12	none	*	NAD	*	ND	*	*	*	*	*	*	0	--
C16	02.10.13	Debre Brehan 16	none	*	Increased respiration rate and difficulty in breathing	*	ND	*	*	*	*	*	*	0	--
C17	14.11.13	Asasa 4	none	*	NAD	*	ND	*	*	*	*	*	*	0	--
C18	14.11.13	Asasa 8	none	*	NAD	*	ND	*	*	*	*	*	*	0	--
C19	14.11.13	Asasa 12	none	*	NAD	*	ND	*	*	*	*	*	*	0	--
C20	14.11.13	Asasa 16	none	*	NAD	*	ND	*	*	*	*	*	*	0	--
Total								25		24		17		25	

NS, no pus or blood sample was collected; ND, = no DNA extract; *, no clinical data or test results; NAD, no abnormalities detected.

Figure S1. Alignment of all 38 cloned fragments of the amplified ITS region showing the 9 consistent SNP's along the 514bp region. The reference sequences of HCC, HCF and HCD were downloaded from Genbank.
